# Protective effects of glucagon‐like peptide‐1 on cardiac remodeling by inhibiting oxidative stress through mammalian target of rapamycin complex 1/p70 ribosomal protein S6 kinase pathway in diabetes mellitus

**DOI:** 10.1111/jdi.13098

**Published:** 2019-07-02

**Authors:** Dongjuan Wang, Longfu Jiang, Beili Feng, Nana He, Yue Zhang, Honghua Ye

**Affiliations:** ^1^ Department of Cardiology Ningbo NO.2 Hospital Ningbo Zhejiang China; ^2^ Stem Cell Laboratory Ningbo No.2 Hospital Ningbo Zhejiang China

**Keywords:** Cardiac remodeling, Diabetes mellitus, Glucagon‐like peptide‐1

## Abstract

**Aims/Introduction:**

Although increased reactive oxygen species (ROS) generation is a major mechanism leading to cardiac remodeling in diabetes mellitus, research into the effects of anti‐oxidation on diabetic cardiac remodeling remains scarce and controversial. Glucagon‐like peptide‐1 (GLP‐1) shows potential anti‐oxidative effects besides lowering blood glucose. The objective of this research was to investigate the effects of GLP‐1 on cardiac remodeling and the molecular mechanism involved in diabetes mellitus.

**Materials and Methods:**

Streptozotocin‐induced diabetic rats received exenatide treatment for 3 months. Cardiac function, cardiac weight index and myocardial interstitial fibrosis were measured. Cardiomyocytes were cultured in high‐glucose medium with GLP‐1 treatment. The ROS production, apoptosis and the levels of mammalian target of rapamycin complex 1/p70 ribosomal protein S6 kinase protein expression in cardiomyocytes were analyzed.

**Results:**

Experimental diabetes mellitus showed impaired cardiac diastolic function, increased brain natriuretic peptide expression and increased interstitial collagen deposition in the myocardium, which were ameliorated by exenatide treatment. Exenatide reduced myocardial ROS production and apoptosis in diabetes mellitus. Also, high glucose‐induced ROS generation and apoptosis in cardiomyocytes were inhibited by GLP‐1, as well as the levels of mammalian target of rapamycin complex 1/p70 ribosomal protein S6 kinase phosphorylation. Furthermore, GLP‐1 treatment upregulated adenosine monophosphate‐activated protein kinase activity in high‐glucose‐induced cardiomyocyte.

**Conclusions:**

Glucagon‐like peptide‐1 protects the cardiomyocytes from oxidative stress and apoptosis in diabetes mellitus, which might contribute to the improvement of cardiac remodeling. The cardiac protection of GLP‐1 might be dependent on inhibition of mammalian target of rapamycin complex 1/p70 ribosomal protein S6 kinase, through an adenosine monophosphate‐activated protein kinase‐mediated pathway.

## Graphical abstract

Glucagon‐like peptide‐1 protects cardiomyocytes against oxidative stress, apoptosis and the resultant myocardial fibrosis in diabetes mellitus, which might contribute to the improvement of cardiac remodeling. The protective effects of glucagon‐like peptide‐1 are dependent on downstream inhibition of mammalian target of rapamycin complex 1/p70 ribosomal protein S6 kinase, through an adenosine monophosphate‐activated protein kinase‐mediated pathway.

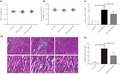

## Introduction

Diabetes mellitus, a common metabolic disease related to many chronic complications, causes a great challenge to public health all over the world^1^. Diabetic cardiomyopathy is a clinical condition of cardiac dysfunction, independent of hypertension, coronary artery disease and cardiac valvulopathy^2^, ^3^. Diabetic cardiomyopathy is a major risk factor for ill health among the diabetic population, and responsible for increased morbidity and mortality^4^. Accumulating evidence has shown that cardiac remodeling, which is characterized by reduced diastolic or systolic function, interstitial fibrosis and pathological left ventricle hypertrophy, plays a key role in diabetic cardiomyopathy^5^. However, there is no effective treatment to prevent cardiac remodeling in diabetes mellitus at present.

Previous evidence showed that oxidative stress in diabetes mellitus was one of the major components in triggering myocardial changes^6^. The increase in reactive oxygen species (ROS) generation subsequently induces apoptosis, inflammation or myocardial collagen deposition^7^. Mammalian target of rapamycin (mTOR), which exists in two structurally distinct complexes, mTOR complex 1 (mTORC1) and mTOR complex 2 (mTORC2), participates in various cellular processes. One recent study showed that mTORC1 played a role in maintaining redox state in diabetes mellitus^8^. Studies in diabetic animals have shown increased mTORC1 phosphorylation in the kidney cortex, and inhibition of the mTORC1 pathway blocked diabetes mellitus‐induced glomerular hypertrophy and albuminuria, which suggested that mTORC1 might relate to cell injury through the oxidative stress pathway^9^.

Therefore, new strategies designed to inhibit ROS production and reduce myocardial mTORC1 expression in diabetes mellitus might prove to be cardioprotective. One such candidate could be glucagon‐like peptide‐1 (GLP‐1), an incretin hormone with many beneficial functions beyond glycemic control^10^, ^11^. We have previously observed that GLP‐1 protected cardiac microvessels from oxidative stress injury in diabetic rats^12^. Also, GLP‐1 analog might have a direct beneficial effect on oxidative stress and diabetic nephropathy through a protein kinase A‐mediated inhibition of renal  nicotinamide adenine dinucleotide phosphate (NADPH) oxidase in diabetic rats^13^. GLP‐1 might be an important contributor to the regulation of whole‐body redox balance. Despite the potential benefits of GLP‐1 in oxidative stress, its cardiac protective effects on diabetes mellitus have been poorly addressed.

Therefore, in the present study, we investigated the protective effects of GLP‐1 on cardiac remodeling in diabetes mellitus, identified whether mTORC1 was involved in the cardiac protection and further characterized the downstream molecular mechanism.

## Methods

### Animals

Two‐month‐old male Sprague–Dawley rats were made diabetic using streptozotocin (Sigma, St. Louis, MO, USA) injection (35 mg/kg) for 3 days^12^. Animals with blood glucose ≥16.6 mmol/L were diagnosed as diabetic, and were randomly divided into two groups: (i) the diabetes mellitus + vehicle group (*n* = 18), which comprised diabetic rats that received saline twice daily for 3 months; and (ii) the diabetes mellitus + exenatide group (*n* = 18), which comprised diabetic that rats received exenatide (0.25 μg/kg; Tocris Bioscience, Minneapolis, MN, USA) twice daily subcutaneously for 3 months. Age‐matched normal animals were used as control group (*n* = 15). To investigate the potential effects of GLP‐1 *in vivo*, GLP‐1 analog exenatide was used in the present study^14^. During the 3‐month treatment period, bodyweight and food intake were recorded weekly and daily, respectively. All animal experiments were carried out under the guidelines on the use and care of laboratory animals for biomedical research published by National Institutes of Health, and approved by Ningbo University Committee on the Ethics of Animal Experiments.

### Blood analysis, intraperitoneal glucose tolerance test and intraperitoneal insulin tolerance test

After the rats fasted overnight, fasting blood glucose, insulin levels, glycated hemoglobin and triglyceride levels were analyzed. Glucose tolerance was assessed by intraperitoneal glucose tolerance test, and insulin tolerance was assessed by intraperitoneal insulin tolerance test. See Appendix S1 for details.

### Cardiac function assessed by echocardiography

Echocardiographic evaluation was carried out using VisualSonics Vevo 2100 (Toronto, ON, Canada) at the end of the experimental period, as described previously^12^. The E/A ratio (E wave, peak velocity blood flow from gravity in early diastole; A wave, peak velocity blood flow in late diastole caused by atrial contraction) was obtained to evaluate the cardiac diastolic function. Two‐dimensional guided M‐mode images from short‐axis view at the level of the papillary muscles were recorded, and left ventricular end‐diastolic diameter, fractional shortening and left ventricular posterior wall thickness at end‐diastole were obtained. All echocardiographic data were analyzed by a single experienced investigator blinded to the groups.

### Cardiac weight index assessment

The cardiac weight index was used to evaluate the hypertrophic response, which was calculated as the heart weight (g) / tibia length (mm)^15^. Briefly, hearts were removed from the rats under anesthesia, rinsed, dried with filter paper and weighed. Tibias from the rats were also isolated and cleaned, then the length of tibias was measured.

### Histological analysis

Masson's trichrome staining was carried out to analyze the collagen fraction in the myocardium^16^. The isolated heart tissues from different groups were prepared and stained with Masson's trichrome. The cardiomyocytes were stained red, whereas the collagen fibers were stained blue. The collagen fraction was evaluated by Image‐Pro Plus 6.0 (Media Cybernetics, Rockville, MD, USA), which was calculated as follows: collagen area / view area × 100%.

### Transmission electron microscopy

The ultrastructure of the myocardium was observed by transmission electron microscope (JEOL JEM‐2000EX, Tokyo, Japan)^12^. Briefly, hearts from anesthetized rats were isolated, and cardiectomy was carried out. Left ventricular tissues were cut into 0.5–1‐mm^3^ pieces, and kept in 4% glutaraldehyde for 24 h and 1% osmium tetroxide for 1 h. Subsequently, the dehydrated tissue samples from different groups were embedded in resin, cut into 60‐nm sections, and stained with uranyl acetate.

### Cultivation and treatment of cardiomyocyte

Cardiomyocytes were isolated from neonatal rats^17^. Hearts were quickly excised and placed into Hank's balanced salt solution. The ventricles were separated from the atria, minced and digested with trypsin for 4–6 cycles. All supernatants were pooled and purified by density centrifugation through a discontinuous Percoll gradient. Cardiomyocytes were collected and cultured in Dulbecco's modified Eagle's medium supplemented with fetal bovine serum (10%). After the cardiomyocytes had been cultured for 4–5 days, the medium was replaced by normal glucose (5.5 mmol/L) or high‐glucose medium (25 mmol/L) with or without GLP‐1 (10^−8^ mol/L). The incubation time of GLP‐1 was 24 h, and the dose of GLP‐1 was based on our previous study^12^.

### Quantification of ROS production

Lucigenin‐enhanced chemiluminescence assay and dihydroethidine staining were carried out to assess ROS generation, as previously described^12^. Data from lucigenin‐enhanced chemiluminescence assay were presented as relative light units per second per mg myocardial tissue weight or relative light units per second per million cells. Dihydroethidine intensity was observed by confocal microscope (Olympus FV 1000, Tokyo, Japan), and analyzed with Image‐Pro Plus 6.0. See Appendix S1 for details.

### Lipid peroxidation measurement

The detection of thiobarbituric acid‐reactive substances (TBARS) levels is a well‐established measure to determine lipid peroxidation^18^. Lipid peroxidation products increase in concentration as a response to oxidative stress. We quantified the levels of TBARS in the cardiomyocytes using a TBARS kit (Cayman Chemical, Ann Arbor, MI, USA).

### Assessment of myocardial apoptosis

Myocardial apoptosis was determined by an In Situ Cell Death Detection Kit (Roche, Penzberg, Germany) following the manufacturer's instructions. The apoptosis rate was calculated as the percentage of terminal deoxynucleotidyl transferase dUTP nick end labeling‐positive nuclei (in green) to the total nuclei (4′,6‐diamidino‐2‐phenylindole, in blue). Sections were immunostained with cardiac troponin T to specifically label the cardiomyocytes.

### Western blot analysis

Protein extraction was carried out as follows: cardiac tissues were isolated, snap‐frozen and extracted in lysis buffer, and cardiomyocytes were harvested at the indicated time and extracted in lysis buffer. The lysis buffer was complemented with phenylmethanesulfonyl fluoride and phosphatase inhibitor. BCA Protein Assay Kit (Thermo Scientific, Rockford, IL, USA) was used to quantify total protein concentration. Protein samples were separated by sodium dodecyl sulfate polyacrylamide gel electrophoresis and transferred onto a polyvinylidene fluoride membrane. The membrane was blocked with 5% skim milk and incubated at 4°C overnight with the following primary antibodies: phospho‐adenosine monophosphate protein kinase (AMPK)‐T172, AMPK, p‐Raptor‐S792, Raptor, p‐mTOR‐S2448, mTOR, p‐p70S6K‐T389, p70S6K, β‐actin (1:1000; Cell Signaling Technology, Danvers, MA, USA) and caspase‐3 (1:200; Santa Cruz Biotechnology, Santa Cruz, CA, USA). The membrane was then incubated with the appropriate horseradish peroxidase‐conjugated secondary antibodies. The blots were developed using an enhanced chemiluminescence system.

### Quantitative reverse transcription polymerase chain reactions

Hearts from each group were isolated, and total messenger ribonucleic acid (mRNA) was extracted with Trizol reagent (Invitrogen, Carlsbad, CA, USA). PrimeScript RT Master Mix kit (Takara, Dalian, China) was used to carry out reverse transcription. Brain natriuretic peptide (BNP) mRNA (primer sequence: forward 5′‐GGGCTGTGACGGGCTGAGGTT‐3′; reverse 5′‐AGTTTGTGCTGGAAGATAAGA‐3′) expression level was detected using Fast SYBR Green Master Mix Kit (Applied Biosystems, Foster City, CA, USA) on fast Real‐Time PCR System (Applied Biosystems), and normalized to β‐actin gene expression. The fold induction of mRNA level was analyzed by the comparative *C*_*t*_ method.

### Small interfering RNA transfection

To suppress Raptor expression, cardiomyocytes were transfected with Raptor‐specific small interfering RNA (siRNA; Santa Cruz Biotechnology) following the manufacturer's instructions. Scrambled siRNA (NC‐siRNA) served as a negative control. After transfection for 24 h, the cardiomyocytes were treated with GLP‐1, and the cell lysates were prepared for further analysis.

### Statistical analysis

Results are presented as mean values ± standard deviation, and analyzed using the anova test followed by Bonferroni's post‐hoc test (apart from western blot data). Western blot results were analyzed with the Kruskal–Wallis test followed by Dunn's post‐hoc test. *P*‐values <0.05 are considered statistically significant. All analyses were carried out using GraphPad Prism 6.0 (GraphPad, San Diego, CA, USA).

## Results

### Basic parameters

During the experimental period, one rat from the control group, two rats from the diabetes mellitus + vehicle group and two rats from the diabetes mellitus + exenatide group died. As shown in Table 1, there was no significant difference in initial bodyweight, blood pressure, heart rate and triglyceride levels among the groups. Compared with the control group, rats from the diabetes mellitus + vehicle group showed high fasting blood glucose (4.7 ± 0.9 mmol/L vs 13.1 ± 1.7 mmol/L, *P* < 0.05), high non‐fasting blood glucose (8.3 ± 2.3 mmol/L vs 22.3 ± 2.5 mmol/L, *P* < 0.05), high glycated hemoglobin levels (4.36 ± 0.29% vs 7.65 ± 0.75%, *P* < 0.05) and decreased insulin levels (2.50 ± 0.38 ng/mL vs 0.56 ± 0.14 ng/mL, *P* < 0.05) after 3 months. However, blood glucose (fasting and non‐fasting), glycated hemoglobin, insulin and triglyceride levels showed little difference between the diabetes mellitus + vehicle group and diabetes mellitus + exenatide group. The diabetes mellitus group showed no statistical difference in glucose tolerance and insulin tolerance compared with exenatide treatment group (Figure S1). In addition, diabetes mellitus rats showed an increase in food intake, whereas exenatide treatment did not affect food intake significantly (Table S1).

**Table 1:** Basic parameters of rats in different experimental groups

Characteristics	Control (*n* = 14)	DM + vehicle (*n* = 16)	DM + exenatide (*n* = 16)
Baseline			
Weight (g)	225.4 ± 7.7	222.8 ± 9.2	221.3 ± 7.1
Heart rate (b.p.m.)	329 ± 11.2	334 ± 10.7	331 ± 14.7
Blood pressure (mmHg)	100.3 ± 5.0	102.5 ± 6.6	103.3 ± 7.4
Fasting blood glucose (mmol/L)	4.6 ± 0.9	11.2 ± 2.3*	11.3 ± 2.4*
Non‐fasting blood glucose (mmol/L)	7.9 ± 1.8	22.1 ± 2.9*	21.9 ± 2.7*
HbA1c (%)	4.11 ± 0.26	4.34 ± 0.26	4.41 ± 0.29
Plasma insulin (ng/mL)	2.55 ± 0.39	0.71 ± 0.10*	0.68 ± 0.13*
Triglyceride (mmol/L)	1.10 ± 0.22	1.06 ± 0.19	1.10 ± 0.20
3 months after treatment			
Weight (g)	437.0 ± 17.1	283.2 ± 21.0*	270.7 ± 23.7*
Heart rate (b.p.m.)	335 ± 10.3	326 ± 15.3	333 ± 14.9
Blood pressure (mmHg)	104.9 ± 8.2	105. 2 ± 8.8	106.7 ± 10.5
Fasting blood glucose (mmol/L)	4.7 ± 0.9	13.1 ± 1.7*	13.5 ± 2.3*
Non‐fasting blood glucose (mmol/L)	8.3 ± 2.3	22.3 ± 2.5*	21.8 ± 3.0*
HbA1c (%)	4.36 ± 0.29	7.65 ± 0.75*	7.49 ± 0.58*
Plasma insulin (ng/mL)	2.50 ± 0.38	0.56 ± 0.14*	0.60 ± 0.12*
Triglyceride (mmol/L)	1.07 ± 0.16	1.53 ± 0.20*	1.46 ± 0.15*

All data are expressed as the mean ± standard deviation.

*P < 0.05 versus control group. DM, diabetes mellitus; HbA1c, glycated hemoglobin.

### Exenatide improved diastolic function in diabetes mellitus rats

**Figure 1:**
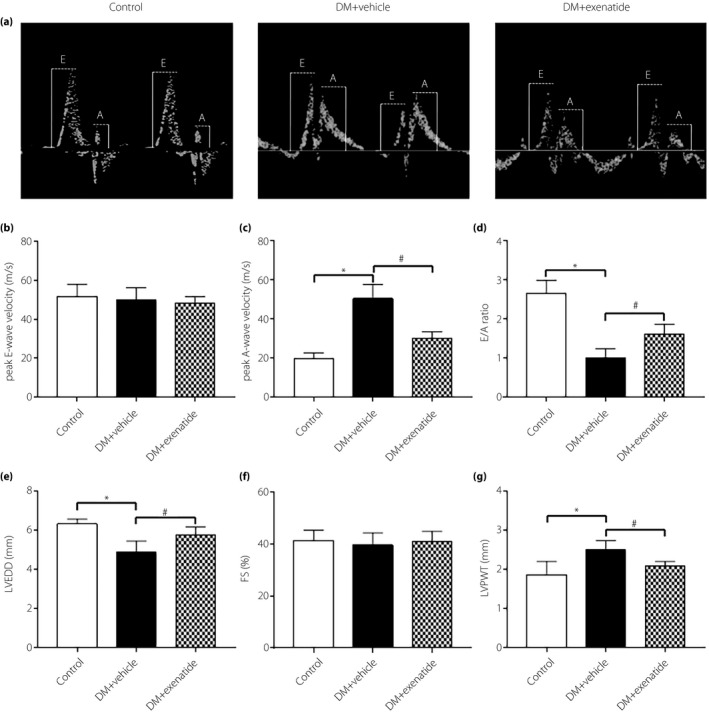
Effects of exenatide on cardiac function in diabetes mellitus (DM) rats. (a) Representative mitral flow patterns from pulsed Doppler. (b) Measurement of peak E‐wave velocity (cm/s). (c) Measurement of peak A‐wave velocity (cm/s). (d) Quantitative analysis of E/A ratio. (e) Measurement of left ventricular end‐diastolic diameter after 3 months of exenatide administration. (f) Measurement of fractional shortening after 3 months of exenatide administration. (g) Measurement of left ventricular posterior wall thickness after 3 months of exenatide administration. Data are expressed as the mean ± standard deviation (*n* = 4–6), *P < 0.05 versus control group, ^#^ *P* < 0.05 versus DM + vehicle group.

After 3 months of exenatide administration, rats from the three groups were anesthetized and echocardiography was carried out to determine the cardiac function. According to Figure 1, diabetic rats showed impaired diastolic function compared with the control group, which manifested as a decreased E/A ratio and left ventricular end‐diastolic diameter (Figure 1a–e). Exenatide treatment for 3 months resulted in an increased E/A ratio and left ventricular end‐diastolic diameter, compared with diabetes mellitus rats treated with vehicle. However, fractional shortening did not statistically differ among the groups investigated (Figure 1f). Furthermore, the results showed that left ventricular posterior wall thickness was slightly higher in diabetic rats than controls, whereas exenatide treatment normalized it (Figure 1g). Taken together, exenatide could improve cardiac diastolic function of diabetic rats.

### Exenatide mitigated cardiac remodeling in diabetes mellitus rats

**Figure 2:**
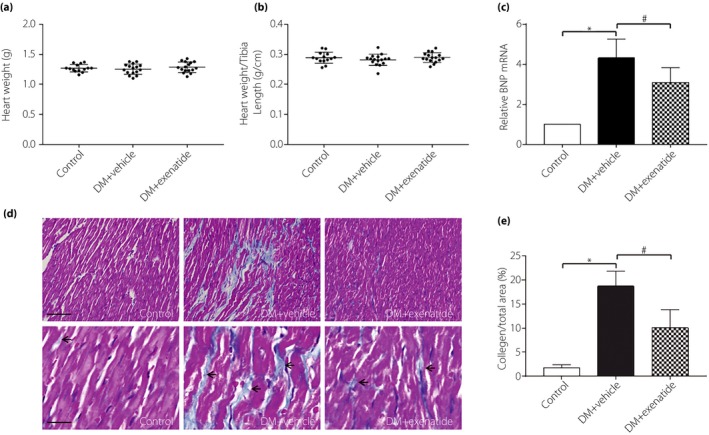
Effects of exenatide on cardiac remodeling in diabetes mellitus (DM) rats. (a) Heart weight (g; *n* = 14–16). (b) Heart weight normalized to tibia length (g/cm; *n* = 14–16). (c) reverse transcription polymerase chain reaction assay for brain natriuretic peptide ( BNP) messenger ribonucleic acid (mRNA) expression in myocardial tissue (*n* = 6). (d) Representative images of Masson's trichrome staining (red, cardiomyocytes; blue, collagen fibers; scale bar of top panel, 10 μm; lower panel, 40 μm). (e) The collagen fraction (as collagen area / view area × 100%) of myocardial tissue (*n* = 5). Data are expressed as mean ± standard deviation, *P < 0.05 versus control group, ^*#*^ *P* < 0.05 versus DM + vehicle group.

Cardiac weight index, BNP mRNA expression and myocardial collagen deposition were measured to determine the role of exenatide on cardiac remodeling in diabetes mellitus. As shown in Figure 2a,b, heart weight and heart weight normalized to tibia length did not differ significantly among the groups. Despite no change in cardiac weight index, BNP mRNA expression was substantially increased in the diabetes mellitus + vehicle group relative to the control group, which was reduced after administration of exenatide for 3 months (Figure 2c). In the normal heart tissue, only a very small amount of extracellular matrix and fibroblasts were observed. Masson's trichrome staining showed that interstitial collagen deposition was increased in the diabetes mellitus + vehicle group, but exenatide led to a significant decrease in collagen deposition in myocardium (Figure 2d,e).

### Exenatide attenuated oxidative stress in heart of diabetes mellitus rats

**Figure 3:**
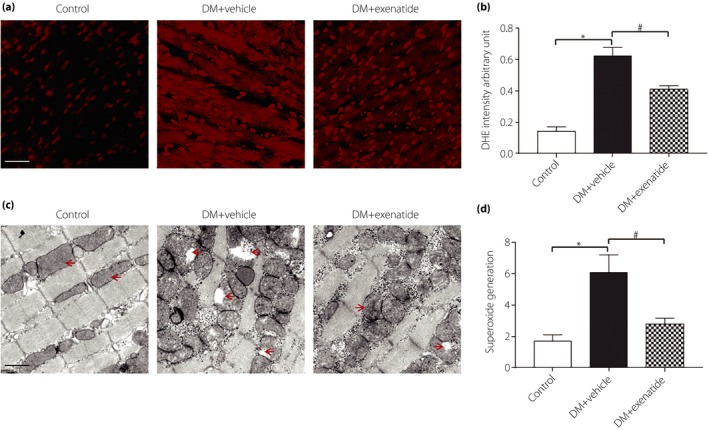
Effects of exenatide on oxidative stress in the heart of diabetes mellitus (DM) rats. (a) Representative images of dihydroethidine (DHE) staining for myocardial tissues (red, DHE; scale bar, 25 μm). (b) The average fluorescence intensity was summarized. (c) Representative electron micrographs for myocardial tissues (mitochondria are indicated by red arrows; scale bar, 1 μm). (d) Superoxide generation (relative light units/s/mg protein) of myocardial tissues from different groups. Data are expressed as the mean ± standard deviation (*n* = 5), *P < 0.05 versus control group, ^*#*^ *P* < 0.05 versus DM + vehicle group.

Reactive oxygen species accumulation was considered to play a key role in diabetic complications^19^, ^20^. Both lucigenin‐enhanced chemiluminescence assay and dihydroethidine staining showed that ROS production trended upward in diabetes mellitus rats, whereas it trended downward after exenatide treatment for 3 months (Figure 3a,b,d). Changes in mitochondrial morphology have been linked to oxidative stress^21^, ^22^, so we further investigated the mitochondria structure using transmission electron microscope (Figure 3c). Mitochondria in myocardium from the control group were aligned in well‐preserved rows between the longitudinally oriented cardiac myofibrils. However, the diabetes mellitus + vehicle group showed significant mitochondrial morphological defects, including disordered mitochondrial arrays, altered cristae density and aggregates of swollen mitochondria, whereas exenatide treatment attenuated the mitochondrial damage in diabetes mellitus. Taken together, these results showed that the increased ROS production promoted in diabetes mellitus could be inhibited by exenatide treatment, concomitant with improved mitochondrial morphology, which suggested that exenatide treatment could attenuate oxidative stress in the heart of diabetes mellitus rats.

### Exenatide decreased myocardial apoptosis in diabetes mellitus rats

**Figure 4:**
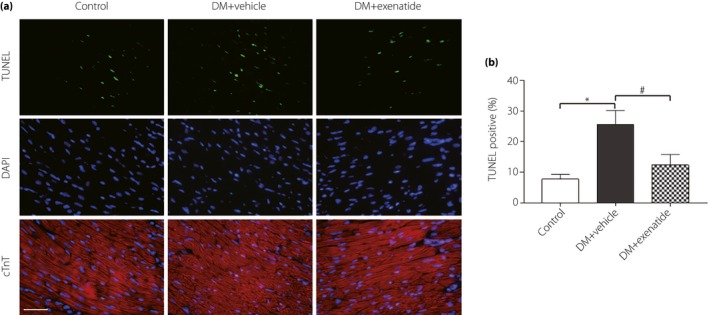
Effects of exenatide on myocardial apoptosis in diabetes mellitus (DM) rats. (a) Representative images of immunofluorescence for apoptotic cells (terminal deoxynucleotidyl transferase dUTP nick end labelling [TUNEL], green). 4′,6‐Diamidino‐2‐phenylindole counterstaining (DAPI; blue) indicates total nuclei and cTn T (cardiac troponin T, red) indicates cardiomyocyte (scale bar: 25 μm). (b) Quantification of apoptotic nuclei by Image‐Pro Plus software. Data are expressed as the mean ± standard deviation (*n* = 5), *P < 0.05 versus control group; ^#^ *P* < 0.05 versus DM + vehicle group.

To further clarify the effects of exenatide on myocardial apoptosis in diabetes mellitus, terminal deoxynucleotidyl transferase dUTP nick end labeling assay was carried out. As shown in Figure 4, compared with the control group, diabetes mellitus rats had a markedly higher rate of myocardial apoptosis. After 3 months of exenatide treatment, the number of terminal deoxynucleotidyl transferase dUTP nick end labeling‐positive cardiomyocytes was significantly reduced.

### mTORC1 involved in the protective effects of exenatide in heart of diabetes mellitus rats

**Figure 5:**
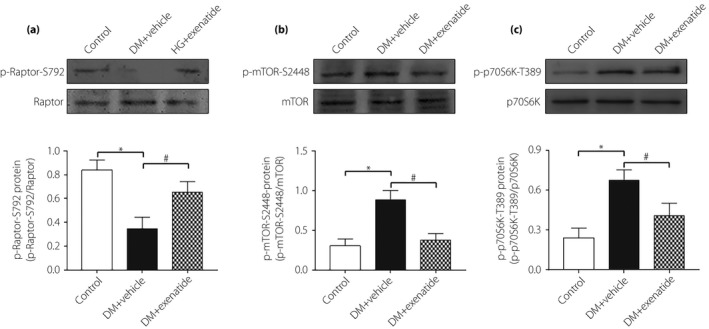
Mammalian target of rapamycin complex 1 (mTORC1) involved in the protective effects of exenatide in the heart of diabetes mellitus (DM) rats. Phosphorylation of (a) Raptor, (b) mTOR and (c) p70S6k in heart tissue measured by western blot. Data are expressed as the mean ± standard deviation (*n* = 3–4), *P < 0.05 versus control group; ^#^ *P* < 0.05 versus DM + vehicle group.

We first investigated the effects of exenatide on the phosphorylation status of protein substrates of the mTORC1 in the heart of diabetes mellitus rats. Western blot showed that the phosphorylation of Raptor was downregulated in diabetes mellitus rats, whereas exenatide treatment upregulated the Raptor phosphorylation. Furthermore, mTOR and p70S6k phosphorylation were increased in the heart tissue of diabetes mellitus rats, which were inhibited by exenatide treatment (Figure 5a–c).

### GLP‐1 reduced high‐glucose‐induced ROS generation through an AMPK/mTORC1/p70S6K‐dependent mechanism in cardiomyocytes

**Figure 6:**
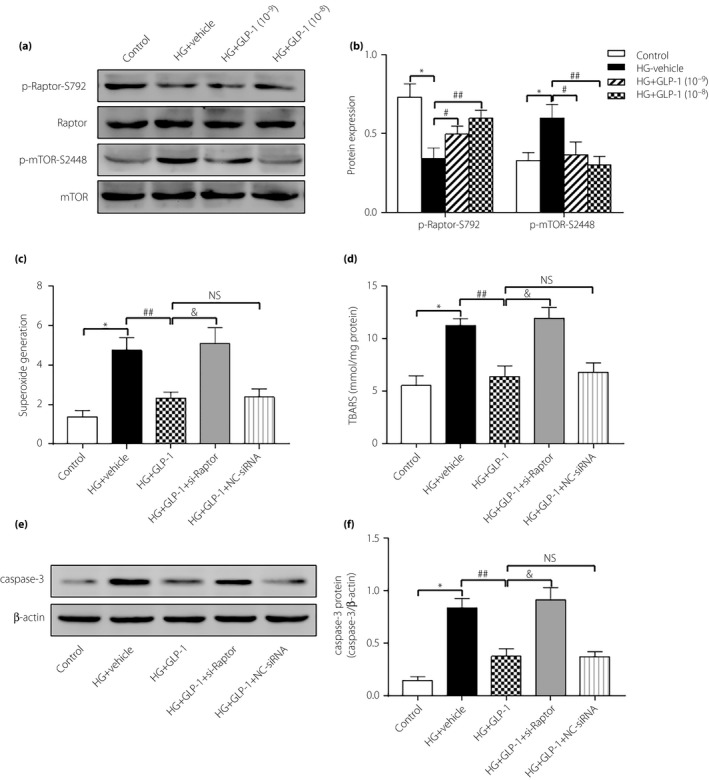
Glucagon‐like peptide‐1 (GLP‐1) suppressed high‐glucose‐induced reactive oxygen species production and apoptosis in cardiomyocytes through mammalian target of rapamycin complex 1 (mTORC1). (a,b) Phosphorylation of Raptor and mTOR in cardiomyocytes measured by western blot. (c) Superoxide generation of cardiomyocytes in different groups. (d) Intracellular levels of thiobarbituric acid‐reactive substances (TBARS) in different groups. (e,f) Cardiomyocytes apoptosis determined by caspase‐3 protein expression. Data are expressed as the mean ± standard deviation (*n* = 3–4), *P < 0.05 versus control group; ^#^ *P* < 0.05 versus high glucose (HG) + vehicle group; ^##^ *P* < 0.01 versus HG + vehicle group; ^&^ *P* < 0.05 versus HG + GLP‐1 group. NS, not significant; GLP‐1 (10^−9^), GLP‐1 concentration at 10^−9^ mol/L; GLP‐1 (10^−8^), GLP‐1 concentration at 10^−8^ mol/L.

To further verify whether mTORC1 is involved in the cardiac protection of GLP‐1, we designed the *in vitro* experiment. Exposure of cardiomyocytes to high glucose markedly downregulated phosphorylation of Raptor and upregulated phosphorylation of mTOR. Treatment of cardiomyocytes with GLP‐1 alleviated the high‐glucose‐induced increase in p‐mTOR expression and decrease in p‐Raptor expression, which was in accordance with the *in vivo* test (Figure 6a,b). It also showed that compared with GLP‐1 treatment at 10^−9^ mol/L, GLP‐1 concentration at 10^−8^ mol/L produced more significant mTOR signaling inhibition.

Raptor siRNA was used to further determine the molecular mechanism behind the beneficial effects of GLP‐1 on cardiomyocytes (Figure S2). Incubation of cardiomyocytes with high glucose promoted intracellular ROS levels, the effects of which were obliterated by GLP‐1 treatment. However, knockdown of Raptor by siRNA before GLP‐1 treatment failed to attenuate ROS production (Figure 6c). We also measured TBARS, a biomarker for oxidative damage to cells, which was increased in high‐glucose‐induced cardiomyocytes, and markedly reduced after GLP‐1 treatment. The production of TBARS was exacerbated in response to treatment with Raptor siRNA (Figure 6d). Along the same line, GLP‐1 was effective in rescuing against high‐glucose‐induced apoptosis, as manifested by caspase‐3 expression in cardiomyocytes (Figure 6e,f).

**Figure 7:**
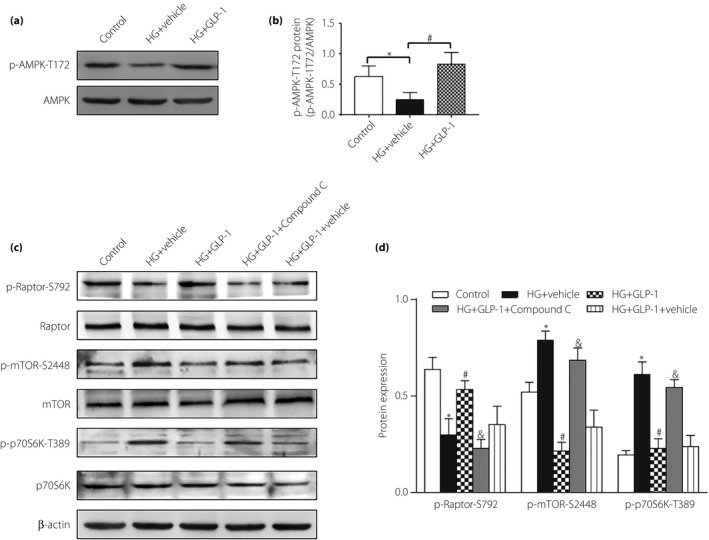
The protective effects of glucagon‐like peptide‐1 (GLP‐1) were through the adenosine monophosphate protein kinase (AMPK)/mammalian target of rapamycin complex 1 (mTORC1)/p70S6K pathway. (a,b) Western blot assay for AMPK phosphorylation. (c,d) Western blot assay for phosphorylation of Raptor, mTOR and p70S6K. Data are expressed as the mean ± standard deviation (*n* = 3–4), *P < 0.05 versus control group; ^#^ *P* < 0.05 versus high glucose (HG) + vehicle group; ^&^ *P* < 0.05 versus HG + GLP‐1 group.

GLP‐1 receptor expression was further detected, which was not found in cultured cardiomyocytes (Figure S3). The cyclic adenosine monophosphate (cAMP) level was also measured (Figure S4). It showed that the cAMP level in the high‐glucose group was increased compared with the control group, whereas GLP‐1 treatment failed to further elevate cAMP level. The results suggested that cAMP was not essential in the GLP‐1/mTORC1 signal pathway in high‐glucose‐induced cardiomyocytes. As AMPK has been reported to be upstream of mTORC1/p70S6K, regulating a number of cellular functions and has specific roles in cardiovascular tissues^23^, we were interested in investigating if AMPK regulated mTORC1/p70S6K in the beneficial effects of GLP‐1 on high‐glucose‐induced cardiomyocytes. We first found that the phosphorylation level of AMPK was decreased in high‐glucose‐induced cardiomyocytes, whereas is was increased after treatment with GLP‐1 (Figure 7a,b). Importantly, the inhibitory effect of GLP‐1 on high‐glucose‐induced phosphorylation of mTOR and p70S6k was abolished by AMPK inhibitor compound C. Also, the phosphorylation level of Raptor was decreased by compound C (Figure 7c,d). Thus, AMPK might probably inhibit the mTORC1/p70S6K pathway and contribute to the protection of GLP‐1 in high‐glucose‐induced cardiomyocytes.

## Discussion

By 2045, diabetes mellitus is expected to affect 693 million people worldwide^24^. Patients with diabetes mellitus have an increased incidence of cardiovascular disease and experience worse clinical outcomes following cardiovascular disease^25^, ^26^. Diabetic cardiomyopathy is a process of relentless progressive cardiac remodeling, which affects approximately 12% of diabetes mellitus patients; however, there is no efficient treatment at present^27^.

It has been reported that in addition to its glucose‐lowering property, GLP‐1 also shows potential cardioprotective properties^28^. Exenatide is the first incretin mimetic, which shares many biological functions with GLP‐1 and has been approved for the treatment of diabetes mellitus clinically^29^. Pretreatment with GLP‐1 protected the heart against ischemic left ventricular dysfunction and improved the recovery of function during reperfusion^30^. Also, GLP‐1 treatment could suppress adverse cardiac remodeling after myocardial infarction^31^. In this sense, it is meaningful to investigate the effects of GLP‐1 on diabetic cardiac remodeling and the molecular mechanism. This research provides the first evidence that GLP‐1 treatment attenuated cardiac remodeling in diabetes mellitus through an AMPK/mTORC1/p70S6K‐dependent mechanism.

Because cardiac dysfunction in type 2 diabetes mellitus has been linked to insulin resistance and the coexistence of obesity, dyslipidemia or hypertension, type 1 diabetes mellitus was used in the present study. The results from this study showed that diabetes mellitus rats for 3 months showed impaired diastolic function, but without systolic function change, and exenatide administration was capable of improving cardiac diastolic function. Several lines of evidence have shown that left ventricular diastolic dysfunction represents the earliest preclinical manifestation of diabetes mellitus, preceding the appearance of systolic dysfunction^32^, ^33^, ^34^.

Interstitial fibrosis was an important mechanism underlying the pathogenesis of diabetes mellitus, which could aggravate the myocardium stiffness, and eventually led to cardiac diastolic dysfunction^35^, ^36^. The present results showed that exenatide administration led to a significant reduction in collagen deposition in diabetes mellitus rats. There was no change in cardiac weight index among the groups. However, the myocardial hypertrophy marker, BNP, was actually increased in diabetes mellitus rates. Thus, it is suggested that the increased extracellular matrix volume could be offset by cardiomyocyte loss, rendering heart weight unchanged in diabetes mellitus rats.

Diabetes mellitus‐associated cardiac dysfunction has been documented to relate to oxidative stress, and subsequently myocardial apoptosis^20^, ^37^. We observed that the myocardial ROS production trended downward after 3 months of exenatide treatment in diabetes mellitus rats. It has been previously shown that mitochondria are especially sensitive to oxidative stress, and mitochondrial defects might further aggravate oxidative stress^38^, ^39^. The present results showed that the myocardial tissue from diabetes mellitus rats exhibited significant mitochondrial morphological defects, whereas exenatide administration attenuated the mitochondrial damage, which provided further support for the inhibitory effects of GLP‐1 on oxidative stress in diabetes mellitus. This investigation also showed that exenatide administration reduced the myocardial apoptosis in diabetes mellitus, which might be associated with a decrease in oxidative stress, and fibrotic infiltration could occur in association with myocyte loss.

mTORC1, relying on the regulatory associated protein (Raptor) of mTOR, has been shown to play a role in cellular oxidative stress of many diseases^40^. Eid *et al*.^41^ reported that in type 1 diabetes mellitus, activation of mTORC1/p70S6K enhanced oxidative stress, and subsequently induced podocyte apoptosis, whereas inhibition of mTORC1 decreased podocyte apoptosis, and attenuated mesangial expansion and albuminuria. The present data showed that exenatide administration reduced mTORC1/p70S6k phosphorylation in heart of diabetes mellitus rats. An *in vitro* experiment also showed that GLP‐1 treatment alleviated the high‐glucose‐induced increase in p‐mTOR expression and the decrease in p‐Raptor expression in cardiomyocytes. We further found that GLP‐1 inhibited oxidative stress and apoptosis induced by high glucose in cardiomyocytes, the effects of which were offset by si‐Raptor pretreatment. These findings showed that high glucose led to oxidative stress and consequent apoptosis in cardiomyocytes, which mediated the progression of cardiac remodeling, whereas GLP‐1 exerted its protective effects through inhibition of the mTORC1/p70S6K pathway.

It is noteworthy that the upstream molecules and mechanisms responsible for GLP‐1‐associated cardiac protection in diabetes mellitus need to be elucidated. In the present study, GLP‐1 receptor expression was not detected in cultured cardiomyocytes, which was in line with the previous reports^42^. The present findings showed that GLP‐1 might directly interact with cardiomyocytes or might be through some unknown receptors. It is true that many cell types, such as endothelial cells, express a functional GLP‐1 receptor, and GLP‐1 receptor signaling involves activation of adenylyl cyclase and increases cAMP generation. However, the protective effects of GLP‐1 unrelated to the GLP‐1 receptor have reported by an increasing number of studies. Husain *et al*. reported that cardioprotective actions of GLP‐1 were preserved in GLP‐1 receptor (−/−) mice, which suggested functional implications of the signal pathway independent of the known GLP‐1 receptor^43^. Shigeto *et al*.^44^ observed little or no cAMP and protein kinase A activation directly in response to picomolar amounts of GLP‐1.

A functional link between AMPK and mTORC1 has been documented^45^, ^46^. It is reported that AMPK exerts its protective actions by inhibiting the mTORC1 pathway in cardiac hypertrophy induced by pressure overload^47^. It is also reported that 4‐hydroxy‐trans‐2‐nonenal could stimulate protein synthesis by activation of the mTORC1/p70S6K signaling pathway in adult cardiac myocytes, most likely mediated by direct inhibition of AMPK^48^. Therefore, we postulated that activation of AMPK might be involved in the GLP‐1‐offered protective effects. In the present study, our results showed that GLP‐1‐induced suppression of high glucose‐induced mTORC1/p70S6K phosphorylation was abrogated when the AMPK pathway was inhibited, which suggested that the cardiac protection of GLP‐1 might be through the AMPK/mTORC1/p70S6K pathway.

The present study provides both *in vivo* and *in vitro* evidence supporting the protective effects of GLP‐1 against oxidative stress through the mTORC1/p70S6k pathway in diabetes mellitus; however, the involvement of the upstream molecular mechanisms was not completely elucidated. It is noteworthy that our findings were mainly based on rodent models with type 1 diabetes mellitus. The cardiac protection and mechanism of GLP‐1 on type 2 diabetes mellitus with cardiovascular disease remain to be determined. Also, caution must be taken in evaluating the cardiac protection of GLP‐1 in diabetes mellitus patients.

In conclusion, we provide evidence that GLP‐1 protects the cardiomyocytes from oxidative stress and apoptosis in diabetes mellitus, which could contribute to the improvement of cardiac remodeling. The cardiac protection of GLP‐1 might be dependent on inhibition of mTORC1/p70S6K, through an AMPK‐mediated pathway. These findings should provide important implications for diabetes mellitus patients, where pharmacological intervention targeting on GLP‐1 might hold promise for prevention and treatment.

## Disclosure

The authors declare no conflict of interest.

## Supporting information


**Figure S1** Glucose tolerance and insulin tolerance in each group.

**Figure S2** Raptor expression was inhibited in cultured cardiomyocytes using si‐Raptor.

**Figure S3** Immunofluorescence and western blot showed the absence of glucagon‐like peptide‐1 receptor expression in cardiomyocytes.

**Figure S4** Effects of glucagon‐like peptide‐1 on cyclic adenosine monophosphate level in high‐glucose‐induced cardiomyocytes.

**Appendix S1** Supplementary materials, methods and results.
**Table S1** Food intake (g/day) in different experimental groups.
