# Supplementary figures and images for: Protective effects of glucagon‐like peptide‐1 on cardiac remodeling by inhibiting oxidative stress through mammalian target of rapamycin complex 1/p70 ribosomal protein S6 kinase pathway in diabetes mellitus
Source: J Diabetes Investig. 2019 Jul 2;11(1):39–51. doi: 10.1111/jdi.13098 (PMC6944832; doi:10.1111/jdi.13098)

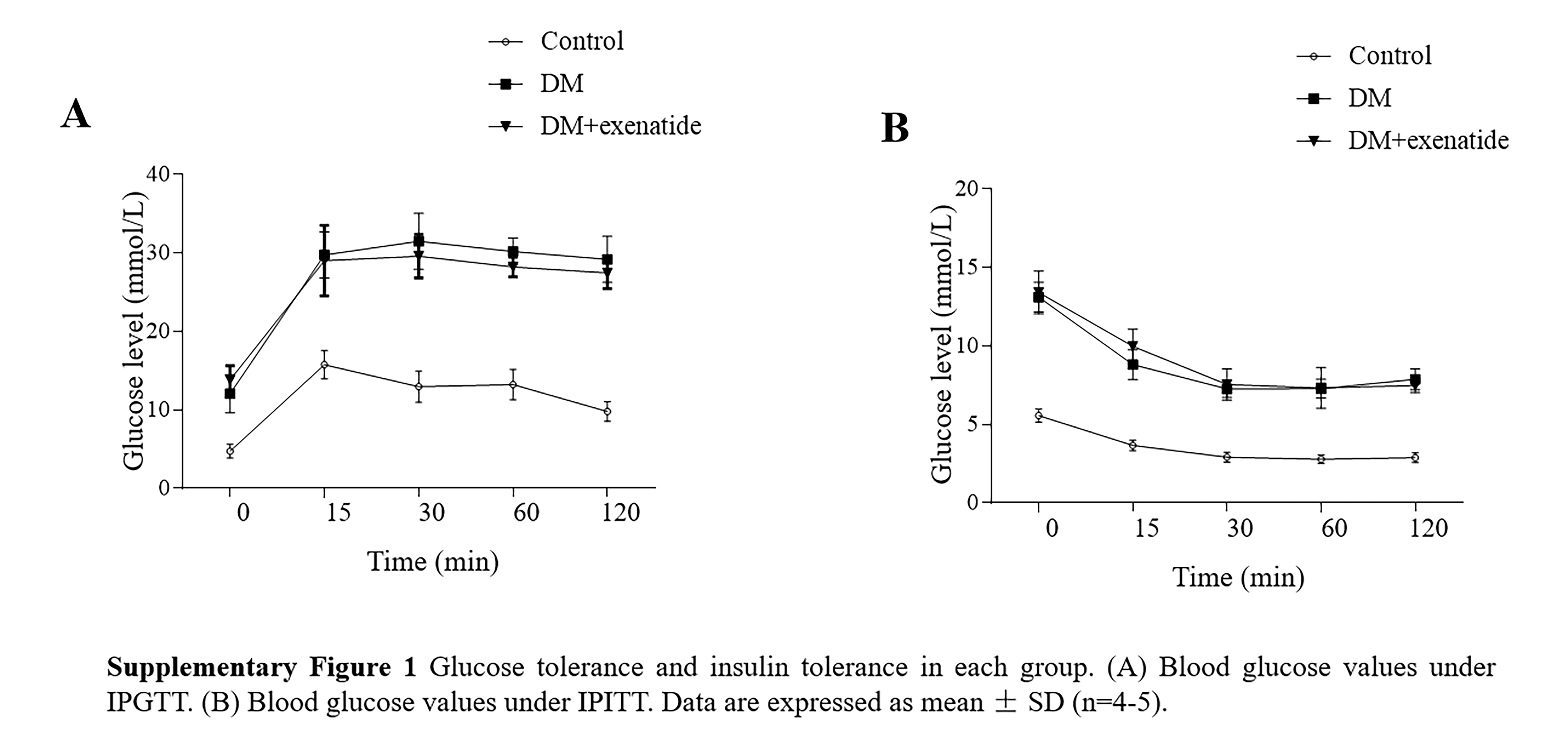

Supplement: Supplementary file 1 — Figure S1 Glucose tolerance and insulin tolerance in each group. [file JDI-11-39-s001.tif]

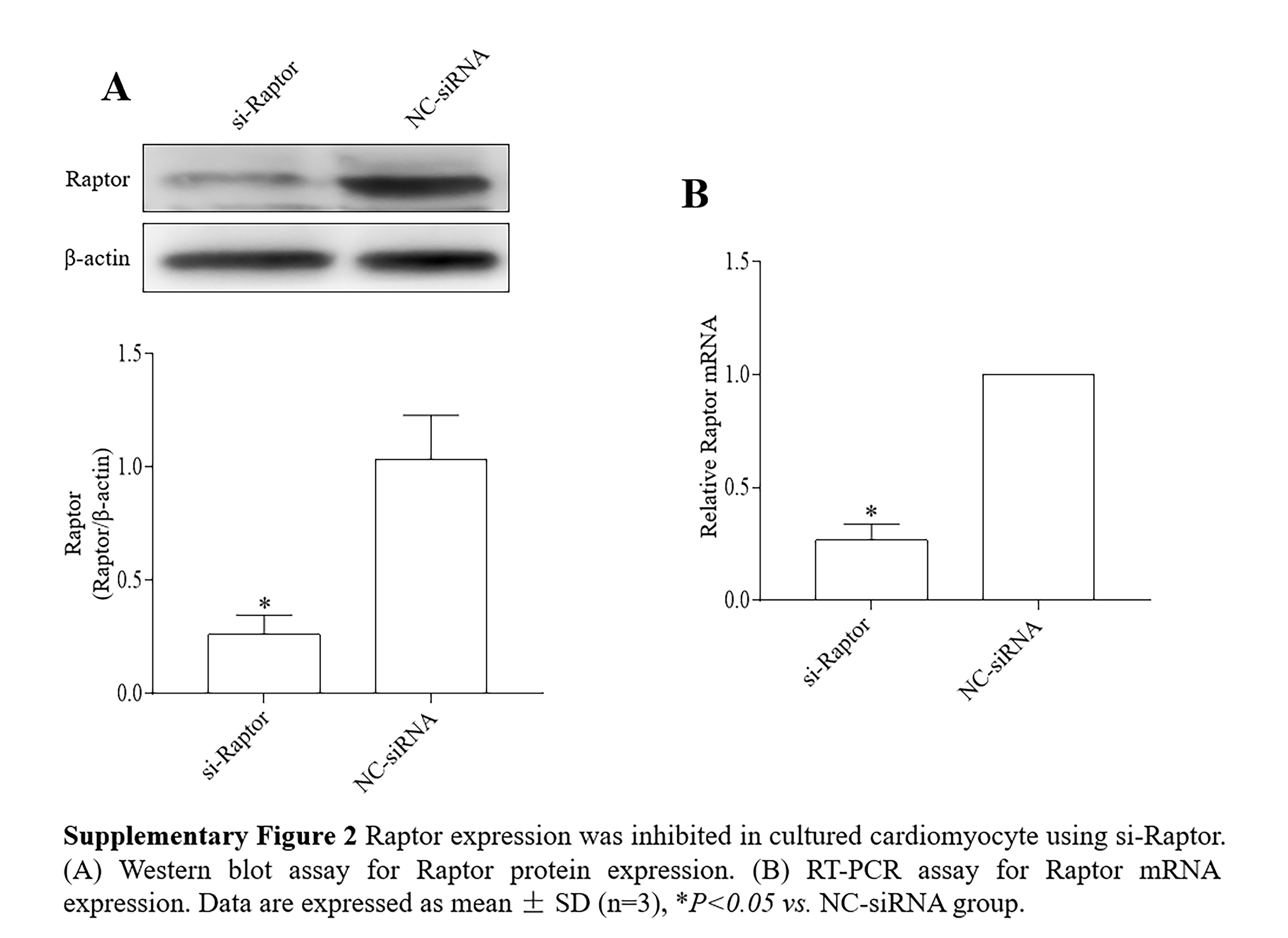

Supplement: Supplementary file 2 — Figure S2 Raptor expression was inhibited in cultured cardiomyocytes using si‐Raptor. [file JDI-11-39-s002.tif]

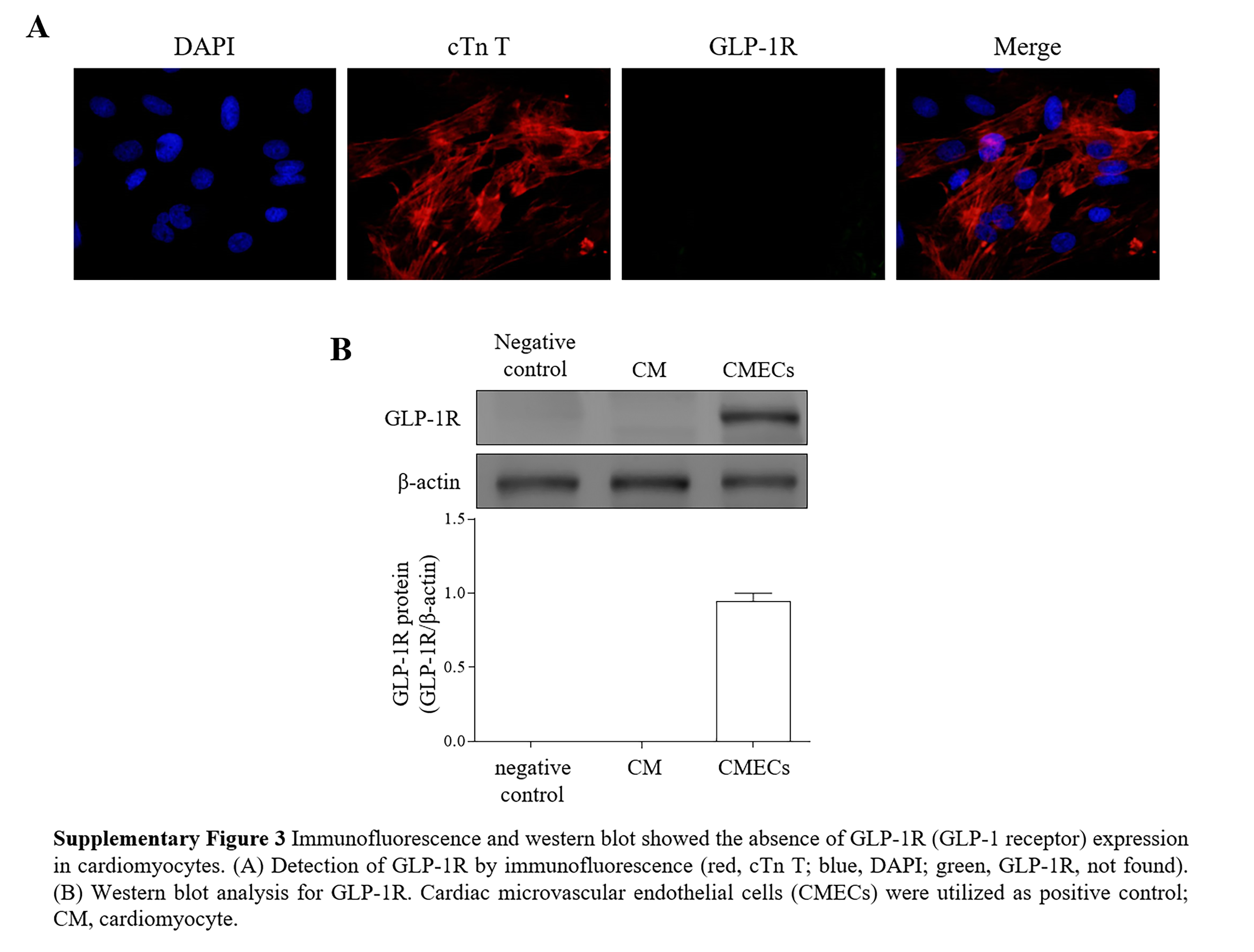

Supplement: Supplementary file 3 — Figure S3 Immunofluorescence and western blot showed the absence of glucagon‐like peptide‐1 receptor expression in cardiomyocytes. [file JDI-11-39-s003.tif]

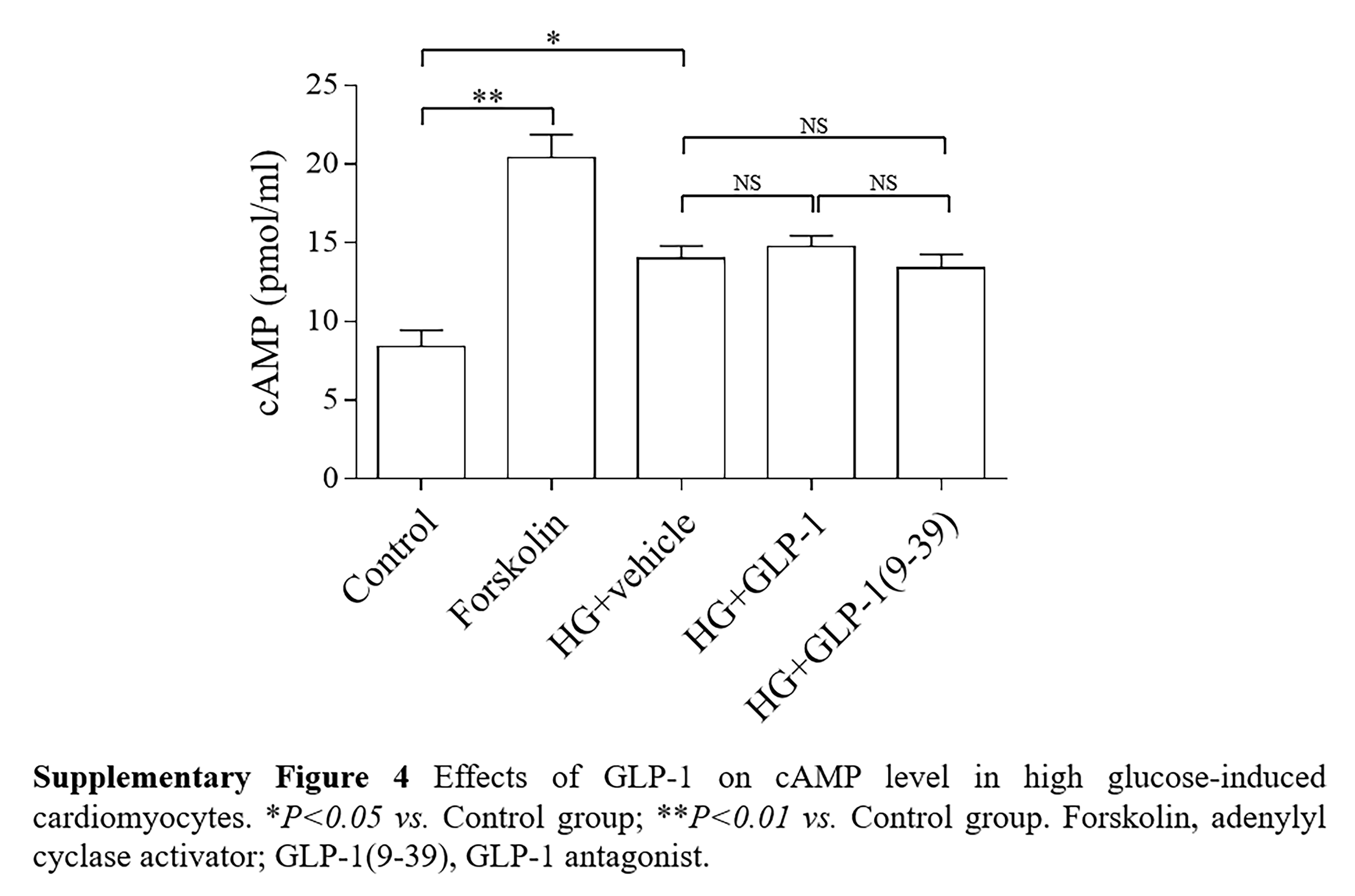

Supplement: Supplementary file 4 — Figure S4 Effects of glucagon‐like peptide‐1 on cyclic adenosine monophosphate level in high‐glucose‐induced cardiomyocytes. [file JDI-11-39-s004.tif]
