# Supplementary material for: Protective effects of glucagon‐like peptide‐1 on cardiac remodeling by inhibiting oxidative stress through mammalian target of rapamycin complex 1/p70 ribosomal protein S6 kinase pathway in diabetes mellitus
Source: J Diabetes Investig. 2019 Jul 2;11(1):39–51. doi: 10.1111/jdi.13098 (PMC6944832; doi:10.1111/jdi.13098)
Supplement: Supplementary file 5 — Appendix S1 Supplementary materials, methods and results. Table S1 Food intake (g/day) in different experimental groups. [file JDI-11-39-s005.docx]

**Supplementary Materials**

**Materials and Methods**

**Blood analysis**

After rats fasted overnight, fasting blood glucose, insulin levels, HbA1c and triglyceride levels were analyzed. Glucose levels were measured by Glucometer (LifeScan, Milpitas, CA), and insulin levels were quantified using insulin ELISA kits (Cusabio Biotech Co., China). In addition, HbA1c and triglyceride levels were analyzed respectively by commercial available assay kits (Nanjing Jiancheng Bioengineering Institute, China), according to the manufacturer's instructions.

**Intraperitoneal glucose tolerance test** **(IPGTT)**

Glucose tolerance was assessed by IPGTT after animals fasted for 12 h. Rats received an intraperitoneal injection of glucose (2 g/kg), blood samples were collected sequentially from tail vein at 0, 15, 30, 60 and 120 min, and glucose levels were determined. The area under the receiver operating characteristic curve was calculated.

**Intraperitoneal insulin tolerance test (IPITT)**

To evaluate insulin tolerance, IPITT was performed after rats fasted for 12 h. Rats received an intraperitoneal injection of insulin (1 unit/kg), and blood samples were taken for glucose measurement at 0, 15, 30, 60 and 120 min. The area under the receiver operating characteristic curve was calculated.

**Quantification of ROS production**

Lucigenin-enhanced chemiluminescence assay and Dihydroethidine (DHE) staining were performed to assess ROS generation. Lucigenin-enhanced chemiluminescence assay was performed as follows: (1) Cells were gently scraped and centrifuged at 2000 rpm for 10 min at 4°C. Then the cell pellet was resuspended in HEPES-buffered saline (140 mM NaCl, 5 mM KCl, 0.8 mM MgCl_2_, 1.8 mM CaCl_2_, 1 mM NaHP0_4_, 25 mM HEPES, and 1% Glucose, PH 7.0). 1×10^6^ cells in suspension were mixed with lucigenin (0.5 mmol/L final concentration), and appropriate blanks and controls were established, then chemiluminescence was recorded with a luminometer (GloMax 20/20, Promega, USA). Chemiluminescence was continuously measured for 12 min, and the ROS production was expressed as relative light units (RLU) per second per million cells (RLU/s/million cells). (2) Left ventricular tissue (30 mg) from each group was placed in ice-cold (4℃) HEPES-buffered saline and homogenized using a pre-cooled homogenizer. The homogenate was then incubated for 30 min at 37℃ in HEPES-buffered saline containing lucigenin (0.5 mmol/L final concentration), and chemiluminescence was recorded with a luminometer. The ROS production was expressed as RLU per second per mg tissue weight (RLU/s/mg).

DHE staining was performed as follows: Left ventricular sections were fixed with 4% paraformaldehyde (Beyotime, China) for 30 min. Dihydroethidium (5 mmol/L in DMSO) was added and incubated at 37°C for 30 min. Omission of DHE was used as a negative control. Nuclei were counter-stained with DAPI. DHE staining was visualized under a confocal microscope (Olympus FV 1000, Tokyo, Japan) and the mean fluorescence intensity of each field was expressed as % fluorescence/mm^2^.

**cAMP measurements**

Cardiomyocytes were treated with normal glucose medium (5.5 mmol/L), high glucose medium (25 mmol/L), GLP-1 (10^-8^ mol/L), and GLP-1(9-39) (10^-8^ mol/L). Forskolin (20 μmol/L) served as a positive control. Measurements of intracellular cAMP concentrations were performed using the cAMP assay kit (R&D Systems) following the manufacturer’s instructions. The enzymatic reaction was quantified by measuring absorbance at 450 nm using a standard plate reader.

**Results**

**Effects of GLP-1 on cAMP level in high glucose-induced cardiomyocytes**

Since GLP-1 affected the promotion of cAMP level in many cell types, we investigated whether cAMP is essential in the GLP-1-mTORC1 signal pathway in high glucose-induced cardiomyocytes. As shown in Supplementary Figure 4, the cAMP level in high glucose group was increased compared with control group, while GLP-1 treatment failed to further elevate cAMP level. There was no significant difference between GLP-1 treatment and GLP-1(9-39) treatment.

**Supplementary Table 1.** Food intake (g/d) in different experimental groups.

|  | Control | DM+vehicle | DM+exenatide |
| --- | --- | --- | --- |
| Month-1 | 22.6±1.9 | 42.8±3.6* | 42.3±2.8* |
| Month-2 | 23.4±2.5 | 40.9±4.1* | 40.2±3.9* |
| Month-3 | 23.9±2.1 | 39.2±3.5* | 40.5±3.0* |

**Legend:** All data are expressed as mean ± SD, ^*^*P<0.05 vs.* Control group.
